# Multi-Stimuli-Responsive Fluorescent Molecule with AIE and TICT Properties Based on 1,8-Naphthalimide

**DOI:** 10.3390/nano14151255

**Published:** 2024-07-27

**Authors:** Yan Yu, Na Qiang, Zhu Liu, Ming Lu, Yuqiu Shen, Jiao Zou, Jinyu Yang, Guocong Liu

**Affiliations:** School of Chemistry and Materials Engineering, Huizhou University, Huizhou 516007, China; yuyan2021@hzu.edu.cn (Y.Y.); qiangna93@hzu.edu.cn (N.Q.); lz0927@hzu.edu.cn (Z.L.); reggi_lu@hzu.edu.cn (M.L.); shenyq666@hzu.edu.cn (Y.S.); zoujiao@hzu.edu.cn (J.Z.)

**Keywords:** stimuli-responsive fluorescent materials, 1,8-naphthalimide, AIE, TICT

## Abstract

A multi-stimuli responsive fluorophore, named NBDNI, was developed by constructing a 1,8-naphthalimide derivative in which a rotatable electron-donating N,N-dimethylaniline group attached to its 4-position. This molecular structure endowed NBDNI with aggregate-induced emission (AIE) and twisted intramolecular charge transfer (TICT) properties, enabling remarkable fluorescence changes in response to multiple external stimuli: (i) sensitivity to polarity in various solvent systems and polymer matrix; (ii) significant fluorescence response and excellent linearity towards temperature changes in solution; (iii) distinct switch of fluorescence color upon acid and base treatments; (iv) reversible mechanochromism behavior in the solid state. Moreover, the mechanisms underlying the aforementioned stimuli-responsive phenomena have been proposed based on comprehensive systematic measurements. Furthermore, preliminary applications such as fluorescence thermometry and acid/base test paper have been demonstrated. This research will bring about new opportunities for the development of novel stimuli-responsive luminescent materials.

## 1. Introduction

Stimuli-responsive fluorescent materials have garnered significant attention due to their ability to exhibit changes in emission behavior in response to external stimuli, such as ions, polarity, pH, temperature, irradiation, and mechanical force [1,2,3,4,5,6,7,8]. These “smart” fluorescent materials hold great promise for applications in chemosensors, data security protection, biological imaging, and so on [9,10,11,12,13,14,15]. Over the past few decades, an increasing number of vibrant fluorophores have been utilized for the construction of stimuli-responsive fluorescent materials. Among them, 1,8-naphthalimide derivatives (NIs) have attracted considerable interest from researchers due to their ease of synthesis, exceptional stability, and high quantum yield [16,17,18,19,20,21]. Importantly, it has been demonstrated that both the N-imide structure of NI and the 4-position of its naphthalene ring can be modified with various electron donor or acceptor groups. As a result of these modifications, the introduction of the twisted intramolecular charge transfer (TICT) effect into NI derivatives’ structures enables them to exhibit colorful emission changes in response to external stimuli [22,23,24,25,26]. However, the development of NIs is hindered by the well-known aggregation-caused quenching (ACQ) effect, which limits the utility of numerous traditional fluorophores in solid-state applications. Fortunately, Tang’s group proposed a successful strategy known as aggregate-induced emission (AIE), which overcomes the ACQ effect by restricting intramolecular motion or other quenching channels within excited fluorophores, resulting in significantly broadened application fields for these fluorophores in real-world scenarios [27,28,29,30,31,32]. Since then, researchers have designed and synthesized various new stimuli-responsive materials based on AIE-active NI derivatives that can function effectively even when aggregated [33,34,35,36,37,38]. For instance, Wang’s research group synthesized a series of temperature-responsive polymers with tuning responsibility towards temperature stimuli in the region of 0–65 °C by using a triphenylamine-naphthalimide chromophore as the signal unit, which can show the red emission in an aggregated state [39]. Moreover, Olson and colleagues reported two 1,8-naphthalimide derivatives that exhibited a reversible fluorescent color change in the solid state to respond to variations in atmospheric relative humidity. Their sensitive emission for water was attributed to the amphiphilic pyridine acceptor and its bromide ion in the 1,8-naphthalimide derivative, which can participate in the emission unit through excited state charge transfer [40]. Recently, Hudson’s research group developed a series of naphthalimide emitters with mechanochromism phenomenon observed in their powder form due to crystalline to amorphous transitions under mechanical force [41]. The disruption of weak intermolecular π–π interactions in the powder form caused by mechanical stimuli led to alterations in fluorescent properties.

**Scheme 1:**
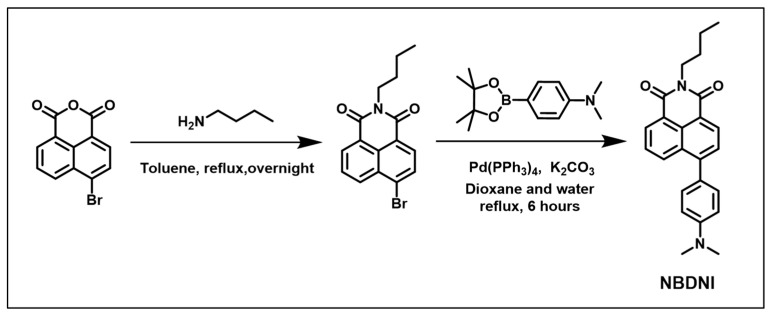
The synthetic route of NBDNI.

Despite these remarkable achievements, the majority of reported stimuli-responsive NI derivatives with AIE have only exhibited single or dual-responsive behaviors. Given the complexity of external applications, it is imperative to develop universal fluorescent molecular sensors, therefore achieving multi-stimuli-responsive fluorescent behaviors with a kind of fluorophore remains a challenge. Herein, we present the design and synthesis of a novel fluorophore named NBDNI based on 1,8-naphthalimide (the chemical structure is shown in Scheme 1). In this molecule, an electron-donor dimethylamino benzene was attached to the 4-position of 1,8-naphthalimide. This simple and ingenious molecular design imparts NBDNI with multiple intriguing fluorescent behaviors upon external stimulation. The photoluminescence properties of NBDNI were thoroughly investigated under different external stimulations, including polarity, acid, temperature, and mechanical force, and these results confirmed its AIE property and multi-stimuli-responsive fluorescent behaviors. Furthermore, possible mechanisms are proposed based on measurement data to elucidate the aforementioned phenomena. We believe that this novel NI derivative could advance understanding of the relationship between fluorophore structures and emission behaviors and enrich the family of stimuli-responsive fluorescent materials.

## 2. Results and Discussion

### 2.1. Synthesis and Characterization

The facile synthetic route to NBDNI is illustrated in Scheme 1, and the detailed synthetic steps are summarized in the supporting information. Here is a concise overview of the procedure. The intermediate was prepared through the reaction between 4-bromo-1,8-naphthalic anhydride and n-butylamine in toluene. Subsequently, NBDNI was obtained as a yellow solid through the Suzuki cross-coupling reaction between the intermediate and 4-(N,N-dimethylamino)phenylboronic acid pinacol ester, followed by further purification. The detailed synthesis processes are depicted in the supporting information, and the chemical structures of all newly synthesized compounds were confirmed using ^1^H NMR or ^13^C NMR spectroscopy (Figure S1).

### 2.2. Polarity Sensitive Fluorescent Behaviors

**Figure 1:**
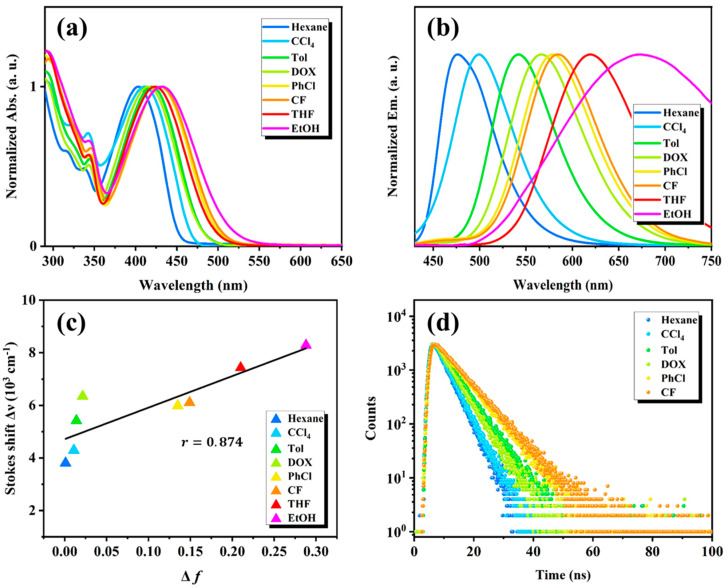
The normalized UV–Vis absorption (**a**) and emission spectra (**b**) of NBDNI (1 × 10^−5^ M) in different solvents, including hexane, tetrachloromethane (CCl_4_), toluene (Tol), 1,4-dioxane (DOX), chlorobenzene (PhCl), chloroform (CF), tetrahydrofuran (THF), and ethanol (EtOH). (**c**) Mataga–Lippert plot of Stokes shift Δν of NBDNI as function of the solvent orientation polarizabilities Δ*f* for these different solvents, and the straight lines represent the linear fits (r = Pearson’s correlation coefficient). (**d**) The fluorescence decay curves of NBDNI in different solvents.

To investigate the solvatochromism behaviors of NBDNI, the photophysical properties of NBDNI in different polarity solvents were measured in detail, and these results are shown and summarized in Figure 1 and Table 1. With increasing solvent polarity, the π–π* absorption peaks were redshifted from 403 nm to 431 nm (Figure 1a). Moreover, a more noticeable redshift and broaden was observed in the emission spectra of NBDNI solutions, and these varied emission peaks from 476 nm to 673 nm, which almost covered the visible light range (Figure 1b). Obviously, the Stokes shift increased with the polarity of solvents, as shown in Figure 1c; the Mataga–Lippert plot for NBDNI gave a positive linear relationship between the Stokes shift (∆ν = ν_abs_ − ν_flu_) and ∆*f*. This interesting solvatochromism behavior is caused by the twisted intramolecular charge transfer (TICT) properties of NBDNI. In the excited state of NBDNI molecules, the rotatable electron donor N,N′-dimethylaminophenyl can undergo twisting relative to the imine ring, causing the NBDNI molecule to form a twisted configuration, known as the TICT state. This twisted configuration is more stable in solvents with higher polarity, resulting in a lower S1 energy level and a narrower energy gap of radiation transition [42,43,44,45]. Therefore, its fluorescence shows more redshift in stronger polar solvents. And conversely, the twisted configuration is unstable in non-polar solvents, so the excited molecules tend to be localized in the plane state, namely the LE state, emitting shorter wavelengths of fluorescence. Meanwhile, in the process of geometric relaxation of excited states for the formation of twisted structures, intramolecular rotation doing work is also beneficial for reducing the S1 energy level. Moreover, in the ground state, the energy level of the planar configuration is lower than the twisted configuration, so the twisted ground state leads to a higher S0 level, which is also conducive to the generation of a narrow energy gap. Lastly, each fluorophore molecule has a different twisting angle and hence emission characteristic, the collection of which thus leads to a wide emission spectrum. The above factors together result in the emission redshift, broadened spectrum. and expanded Stokes shift as the solvent polarity increases.

Furthermore, the quantum yields and lifetimes of NBDNI in different solvents were measured (Table 1 and Figure 1d). The quantum yields of NBDNI solutions exhibited polarity dependence. As the solvent polarity increased, the quantum yield decreased. We believe that this phenomenon is related to the alteration between the LE state and the TICT state of NBDNI caused by solvent polarity. In the TICT state, the electron-donating and electron-withdrawing groups tend to be perpendicular to each other, making it difficult to achieve efficient radiative transitions in the TICT state. In the LE state, the electron-donating and electron-withdrawing groups tend to be coplanar, which can achieve effective molecular orbital overlap and facilitate the occurrence of radiative transitions. As a result, the TICT state caused by strong polar solvents leads to a lower quantum yield, and the LE state in non-polar solvents showed higher values.

**Figure 2:**
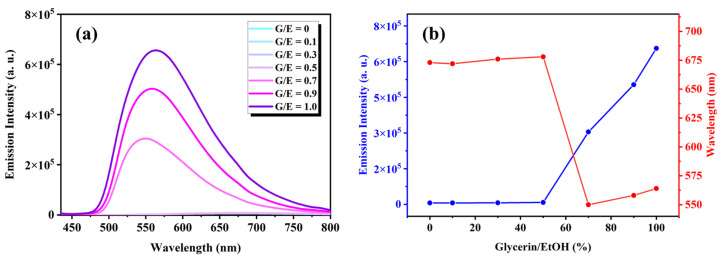
Emission spectra (**a**) and variation of emission intensity and wavelength maximum (**b**) of NBDNI in different ratio of Glycerin/EtOH.

Moreover, we investigated the fluorescence spectra of NBDNI in various ratios of glycerin/ethanol systems to understand the influence of the viscosity environment. As shown in Figure 2a,b, a distinct emission enhanced with blueshift was observed with the increasing content of glycerin in the solution. Due to a serious charge separation in the ethanol solvent, the NBDNI in EtOH solvent demonstrated a very low emission so its quantum yield cannot be determined. However, due to the addition of glycerin into the system, the increasing viscosity inhibited the intermolecular rotation of NBDNI so it was difficult to form a TICT state; further, the LE state led to emission-enhanced intensity and the blueshifted wavelength in a higher viscosity environment.

**Figure 3:**
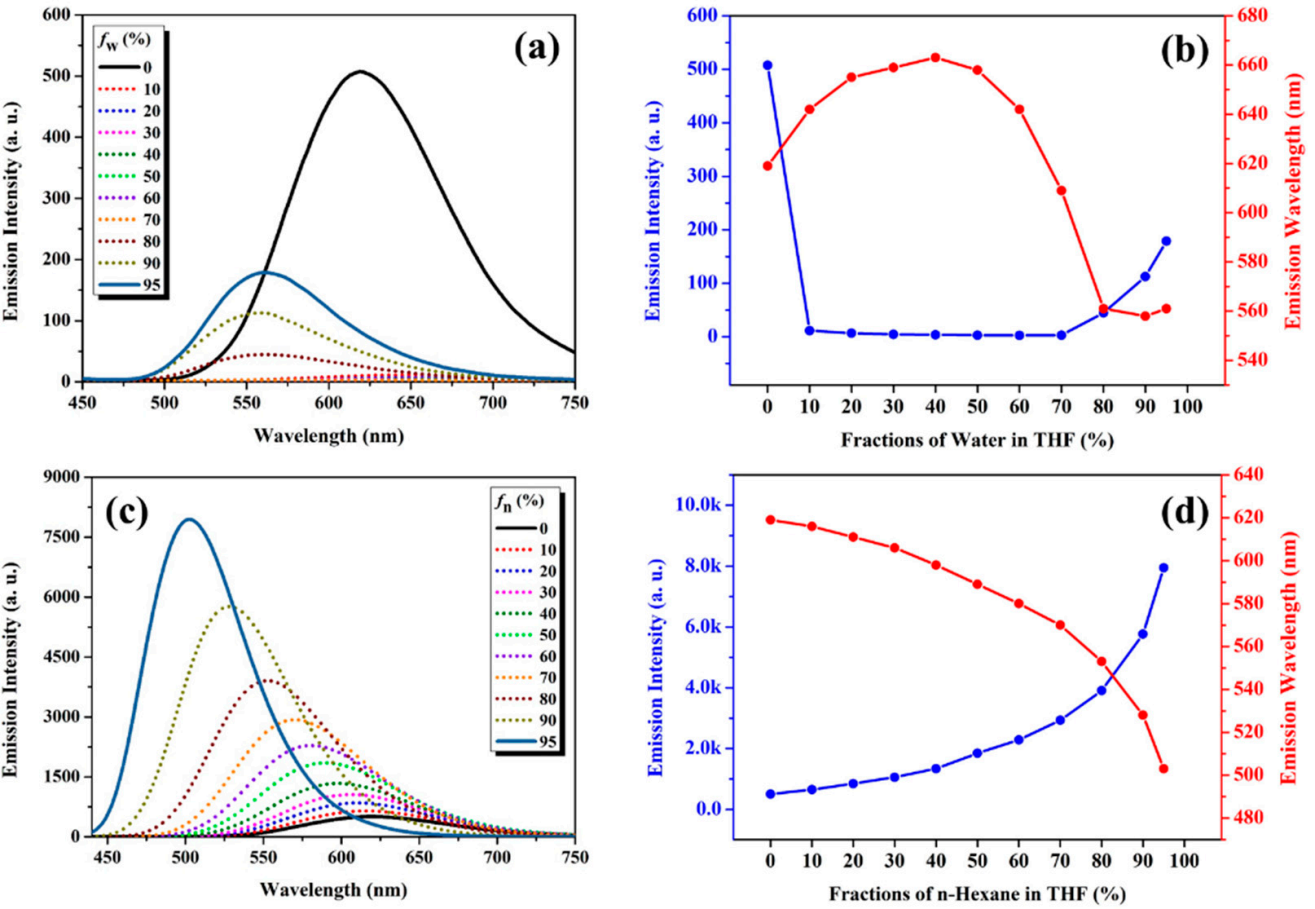
Emission spectra of 1 × 10^−5^ M NBDNI in different ratios of water/THF (**a**) and n-hexane/THF (**c**) excited at 420 nm. Variation of emission intensity and wavelength maximum of 1 × 10^−5^ M NBDNI in different ratios of water/THF. (**b**) and n-hexane/THF (**d**) excited at 420 nm.

To gain a deeper understanding of the polarity-sensitive fluorescence behavior of NBDNI and its dependence on the aggregation state, we investigated the fluorescence properties of NBDNI in various ratios of water/THF solutions and n-hexane/THF solutions. As depicted in Figure 3a,b, in different ratios of water/THF solvents, an increasing water content resulted in a significant decrease followed by a slight recovery in the fluorescence intensity of NBDNI. Simultaneously, there was a gradual redshift followed by a gradual blueshift observed in the emission wavelength over the whole process of increasing water content. This interesting fluorescence behavior was attributed to the combined effects of polarity and solubility on NBDNI. Initially, when dissolved in pure THF, NBDNI exhibited red fluorescence at 620 nm. Upon increasing the water content to 10%, there was a pronounced redshift caused by the TICT effect for enhanced solvent system polarity. Simultaneously, the emission intensity weakened significantly to only 1/44th compared to that observed in pure THF. As the water content reached 40%, a maximum polarity effect induced by water was achieved; therefore, the fluorescence wavelength further redshifted to 663 nm while simultaneously decreasing emission intensity to just 1/160th compared to its initial state. However, as we continued increasing the water ratio, an interesting phenomenon occurred where the fluorescence wavelength turned to blueshift. After reaching 80% water content, NBDNI exhibited blueshifted fluorescence at approximately 580 nm. Additionally, the emission intensity started showing enhancement after surpassing 70% water content. Meanwhile, the increase in water content to 95% resulted in approximately 56-fold fluorescence intensity compared to when water content was at 70%. This phenomenon of blueshifting and enhancement of fluorescence was attributed to the aggregation of NBDNI after the the water content in the system reached a certain level. The phenomenon of fluorescence blueshift and partial recovery mentioned above is attributed to the aggregation of NBDNI upon reaching a certain water content in the system. On one hand, intermolecular aggregation gradually shields the influence of external polarity, resulting in a blueshift of fluorescence. On the other hand, intermolecular aggregation suppresses the intramolecular movement of NBDNI, reducing non-radiative transitions and enhancing emission intensity. In brief, we can conclude that with increasing water content, the fluorescence behavior of NBDNI exhibited two distinct stages. The first stage was dominated by polarity sensitivity arising from its TICT property, while the second stage was primarily induced by its AIE property. The antagonism between these two properties led to a two-stage fluorescence behavior observed in the whole increasing water content process.

Meanwhile, a noteworthy different fluorescence behavior of NBDNI was observed in mixed solvents with varying ratios of n-hexane/THF. As shown in Figure 3c,d, with the gradual increase of n-hexane content, the fluorescence intensity of NBDNI significantly increases. Finally, when the n-hexane content was 95%, its emission intensity reached about 15.7 times that observed in a pure THF solution, accompanied by a significant blueshift of the fluorescence wavelength from 620 nm to 503 nm. This unique fluorescence behavior can be also attributed to the combined effects of polarity and solubility since n-hexane acts as both a non-polar solvent and a poor solvent. On the one hand, when n-hexane was added to the THF solution of NBDNI, it gradually reduced the polarity of the mixed solvent system, leading to a blueshifting fluorescence of NBDNI with TICT property. On the other hand, due to its poor solvency properties, adding n-hexane caused the aggregation of NBDNI molecules, which inhibited non-radiative transitions induced by intramolecular motion and consequently enhanced fluorescence intensity. Overall, in mixed solvents with different n-hexane/THF ratios of NBDNI, with the increasing n-hexane content, the entire fluorescence change was a monotonic process where TICT and AIE properties of NBDNI synergistically influenced this system.

**Figure 4:**
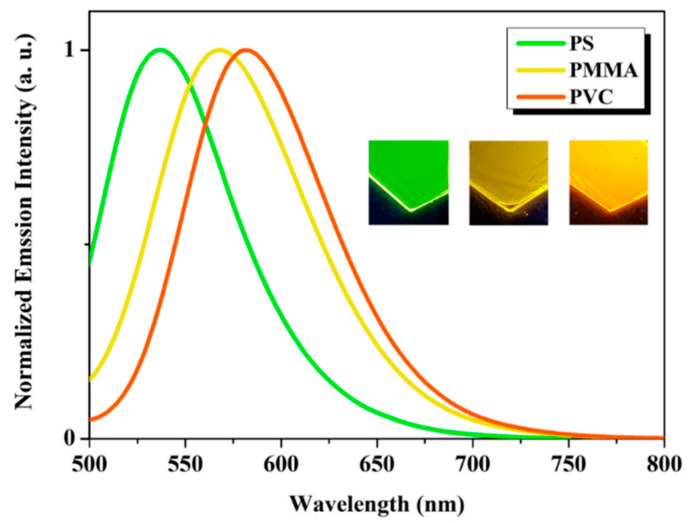
The normalized fluorescence emission spectra of PS, PMMA, and PVC films doped with 5 wt.% NBDNI. The inset is the photo of the above three films upon 365 nm UV light. All of the films were prepared by spin coating on quartz chips.

Furthermore, we investigated how different polar polymers affect the fluorescent emissions from NBDNI molecules by preparing doped polymer films using polystyrene (PS), polymethyl methacrylate (PMMA), and polyvinyl chloride (PVC) as matrices. The corresponding fluorescence spectra are presented in Figure 4 wherein PS-doped film exhibited peak wavelengths at 537 nm while PMMA- and PVC-doped films displayed peak wavelengths at 569 nm and 582 nm, respectively. The inset shows fluorescence photos of three doped films under 365 nm handheld UV light source irradiation. The PS-, PMMA-, and PVC-doped films demonstrated yellow and orange fluorescence, respectively. The above results indicate that NBDNI also exhibits polarity-sensitive fluorescence behavior when dispersed in different polarity polymers in a solid state.

### 2.3. Thermosensitive Fluorescent Behaviors

**Figure 5:**
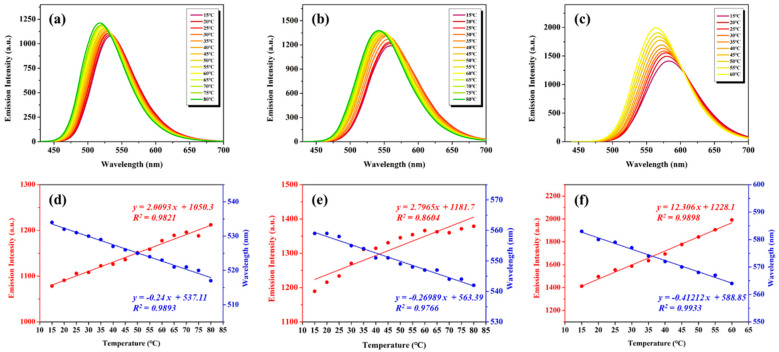
Fluorescent emission spectra of NBDNI (1 × 10^−5^ M) in toluene excited by 420 nm UV light at 15~80 °C (**a**), in dioxane at 15~80 °C (**b**), and in CHCl_3_ at 15~60 °C (**c**). Temperature dependence of emission intensity and maximum emission wavelength of NBDNI (1 × 10^−5^ M) in toluene at 15~80 °C (**d**), in dioxane at 15~80 °C (**e**), and in CHCl_3_ at 15~60 °C (**f**).

Considering the favorable formation of the LE state with increasing temperature and the TICT state with decreasing temperature [3,46,47,48], we studied the photophysical properties of NBDNI in a toluene solution at different temperatures. As is shown in Figure S2, the UV–Vis absorption peak of NBDNI at around 420 nm did not notably change with the increasing temperature. However, a more significant change was observed in the fluorescence emission spectra of NBDNI solutions as the temperature increased. As depicted in Figure 5a, in a toluene solution of NBDNI, the maximum fluorescence emission wavelength was 534 nm at 15 °C. Upon raising the temperature to 80 °C, this wavelength blueshifted to 517 nm while also enhancing the fluorescence intensity by approximately 1.12 times compared to its initial value. Linear fitting analysis revealed that both fluorescence wavelength and intensity of NBDNI exhibited a linear response towards varying temperatures in toluene (Figure 5d). Similarly, as a sample for a polar solvent system, the emission spectra of the NBDNI dioxane solution with varied temperatures were measured, and the corresponding functional relationships were simulated (Figure 5b,e). However, although there was a wider range of changes observed in the emission peak compared to that seen in toluene solution upon increasing temperature, there was not an ideal linear relationship between λ_max_ or I_max_ and temperature. We propose that the increased temperature weakened the hydrogen bonds between dioxane and NBDNI molecules, thereby affecting the intramolecular charge distribution of NBDNI. This effect combined with the temperature dependence of the TICT state leads to deterioration of the linearity between fluorescence emission and temperature variation. Thus, chloroform was chosen as a middle polar solvent without a hydrogen-bond donor for the thermosensitive measurement. As depicted in Figure 5c,f, in the NBDNI chloroform solution, the fluorescence exhibited a maximum emission wavelength of 583 nm at 15 °C, which blueshifted to 564 nm upon reaching a temperature of 60 °C with an accompanying increase in fluorescence intensity by a factor of 1.41. The fitting function between temperature and fluorescence intensity or maximum emission wavelength demonstrated that the temperature-sensitive fluorescence of NBDNI in chloroform displayed significant responsiveness and excellent linearity towards changes in temperature. 

**Figure 6:**
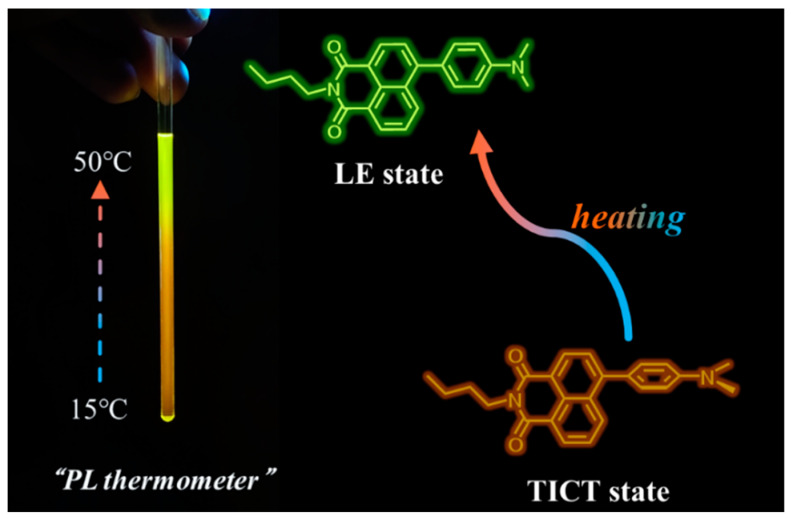
Photograph of fluorescence at different temperatures (15~50 °C) of NBDNI in CHCl_3_ solution in a quartz tube (**left**). The schematic diagram of the mechanism of thermosensitivity of NBDNI molecule (**right**).

In order to have an intuitive understanding of the applicability of NBDNI in the field of temperature-sensitive fluorescence sensing, we employed a simple “fluorescence thermometer” by placing the chloroform solution containing NBDNI into a quartz tube. After heating the upper part of the quartz tube, the temperature of the top part of the solution in the tube reached 50 °C while maintaining a bottom solution temperature of 15 °C. As shown in Figure 6, under irradiation with 365 nm UV light, there was an evident transition from weak orange light to intense green light along the lengthwise axis within the tube, thereby providing clear contrast in both color and brightness corresponding to different temperatures. These results underscored that NBDNI holds immense potential for applications as a fluorescent temperature sensor.

### 2.4. Acid-Triggered Fluorescence Switch

**Figure 7:**
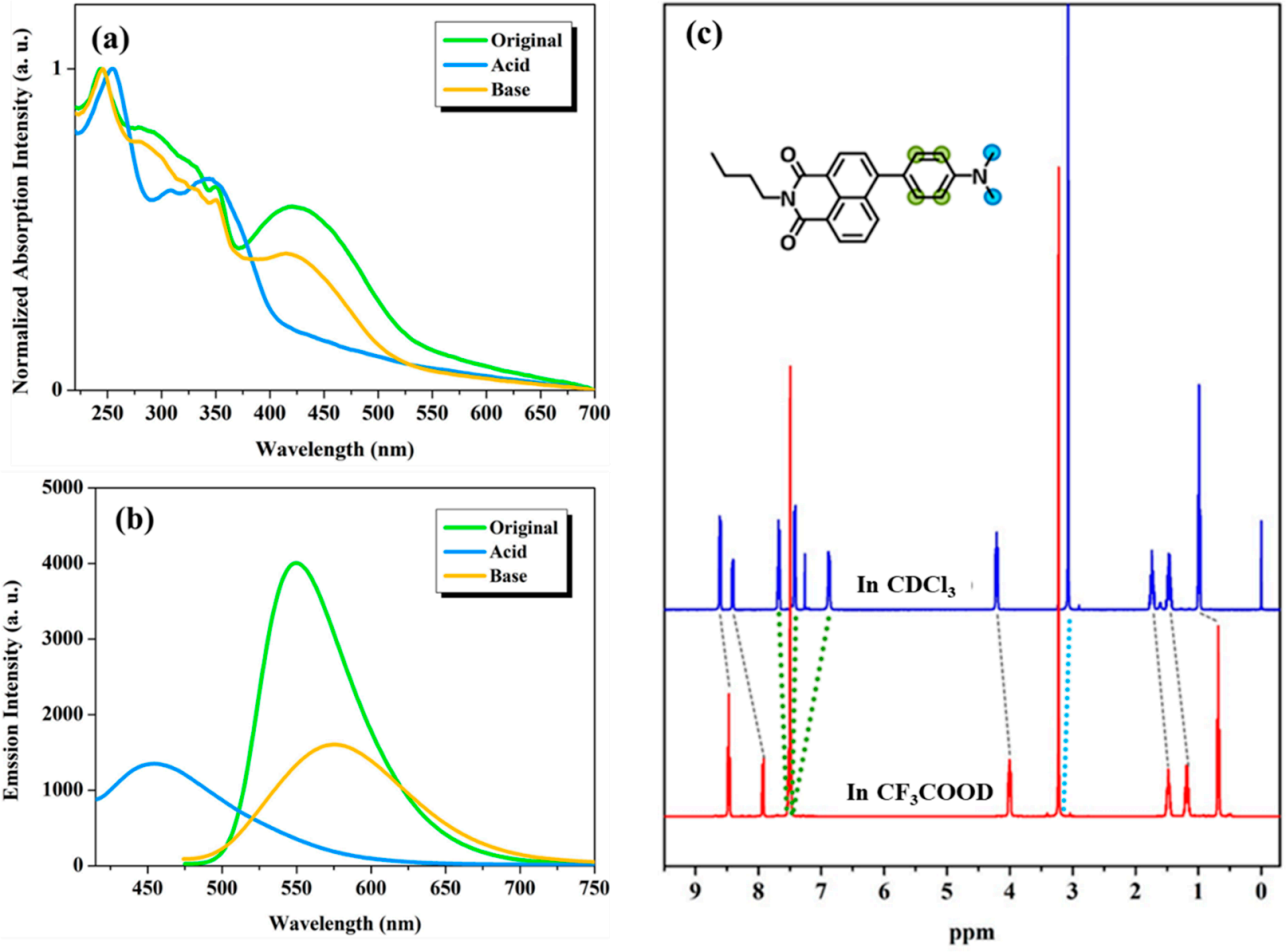
The normalized UV–Vis absorption spectra (**a**) and fluorescence emission spectra (**b**) of original NBDNI powder and its processed sample with acid and base. The ^1^H NMR spectra of NBDNI in CDCl_3_ and CF_3_COOD (**c**).

Considering the existence of the N,N-dimethylamino group in NBDNI, we investigated the fluorescent behaviors of NBDNI under acidic and basic conditions. The as-prepared NBDNI solid powder emitted bright green fluorescence at 546 nm with a 15.06% quantum yield. The NBDNI solid powder was fumed with hydrochloric acid (HCl) and then fumed with triethylamine (TEA). The UV–Vis absorption of NBDNI in the above three states is shown in Figure 7a. After fumigation with hydrochloric acid, the absorption peak at 420 nm disappeared while a new peak emerged at 344 nm. Moreover, after fumigation with triethylamine, the absorption peak at 420 nm was recovered partially while the peak at 344 nm vanished. The changes in absorption spectra under acidic and basic conditions were attributed to protonation and deprotonation of the dimethylamino group in NBDNI, which affects electron distribution in the molecule. Subsequently, fluorescence emission spectra of NBDNI solids were measured for each state, as depicted in Figure 7b. It was observed that the original NBDNI exhibited strong green fluorescence at 546 nm. Upon treatment with hydrochloric acid, this emission transformed into weak blue fluorescence peaking at 454 nm due to a diminished electron-donating ability resulting from protonation of the dimethylamino group. However, following fumigation with triethylamine, yellow fluorescence emission appeared at 576 nm instead of the green light observed initially. This change may be attributed to the neutralization between triethylamine and hydrochloric acid-forming salts, which influenced the fluorescence color of NBDNI as a polarity-sensitive molecule.

In order to better understand the reason for the fluorescence blueshift of NBDNI in the presence of acids, the NMR hydrogen spectra were characterized using deuterated chloroform (CDCl_3_) and deuterated trifluoroacetic acid (CF_3_COOD) as solvents, respectively. As shown in Figure 7c, the peak of the chemical shift at 3.07 ppm in CDCl_3_ moved towards the lower field (sky blue dotted line) in CF_3_COOD, while the peaks at 7.67, 7.42, and 6.87 ppm merged into a single peak at 7.50 ppm (green dotted line). Additionally, the peaks at 8.61, 8.41, 4.21, 1.74, 1.46, and 0.98 ppm moved towards the high field (black dotted line). We propose that these observations indicate the protonation of NBDNI in acidic conditions as well as the formation of hydrogen bonds.

Firstly, the peak at 3.07 ppm in CDCl_3_ was attributed to hydrogen atoms of the dimethylamine group (highlighted in blue in the molecular structure shown in Figure 7c), whereas the protonated nitrogen atom on the dimethylamine group in CF_3_COOD leads to a deshielding effect on the hydrogen atoms of the methyl group; therefore, their chemical shift peak move towards the lower field. Secondly, the protonation weakens the electron-donating ability of dimethylamine group, and the electron-absorbing ability of carbonyl groups on the imide structure was weakened by the hydrogen bonds between the CF_3_COOD and them. This resulted in the destruction of the original D-A structure leading to a more homogeneous distribution of the electron cloud so that the more similar chemical environment of benzocyclic hydrogens (highlighted in green in the molecular structure shown in Figure 7c) caused the merging of the peaks at 7.67, 7.42, and 6.87 ppm. Finally, the weakened electron-withdrawing ability of the carbonyl groups in CF_3_COOD led to an increased shielding effect on the remaining hydrogens in NBDNI, and therefore, their chemical shift peaks moved towards the higher field. These above ^1^H NMR results reflected the existence of protonation and formation of hydrogen bonding in the acid and thereby changed the distribution of electrons in the NBDNI molecule, resulting in the blueshift of its emission.

**Figure 8:**
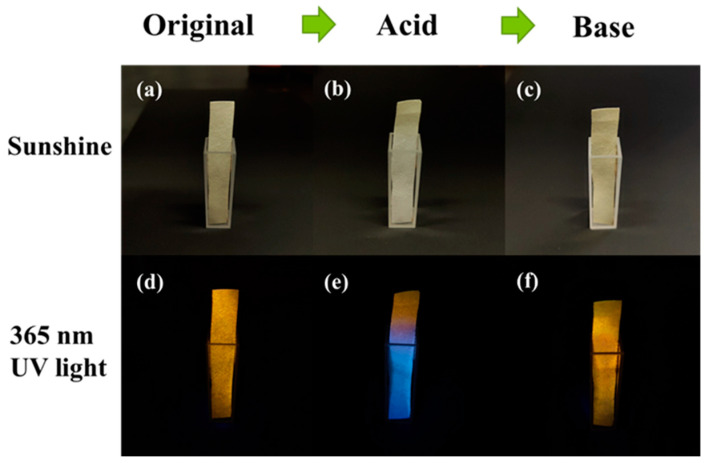
Photographic images of fluorescence test paper in original state, fumed by acid and then fumed by a base, respectively, under sunshine (**a**–**c**) and 365 nm UV light (**d**–**f**). The test paper was prepared by drying the filter paper that was soaked with NBDNI solution.

We also conducted a preliminary exploration of the application of NBDNI’s acid fluorescence responsiveness. As demonstrated in Figure 8, the strip paper was soaked in THF solution containing 1 × 10^−5^ mol/L NBDNI, and after the solvent evaporated, a fluorescent acid test paper was obtained. It is worth noting that unlike the green fluorescence observed in its original as-prepared NBDNI solid powder, the obtained test strip exhibited yellow fluorescence under 365 nm UV light. This phenomenon can be attributed to the fact that recrystallized NBDNI molecules in solid powder form a regular and densely packed structure with planar molecular conformation, predominantly existing in the LE state. However, when prepared in a diluted solution and infiltrated into the paper matrix, these molecules retained their twisted conformation that was formed during the tetrahydrofuran drying process. Upon excitation by ultraviolet light, they entered into the TICT state, leading to redshifted fluorescence from green to yellow emissions. To further investigate this behavior, we initially exposed half of the NBDNI test strip to hydrochloric acid vapor, which resulted in blue fluorescence emission under 365 nm UV light irradiation. Subsequently, treating this acid-treated NBDNI test paper with triethylamine restored its original yellow fluorescence emission again. These results clearly demonstrate that NBDNI exhibits high sensitivity towards external acidic or basic conditions and holds great potential for applications as pH-sensitive fluorescent sensors.

### 2.5. Reversible Mechanochromism Behavior

**Figure 9:**
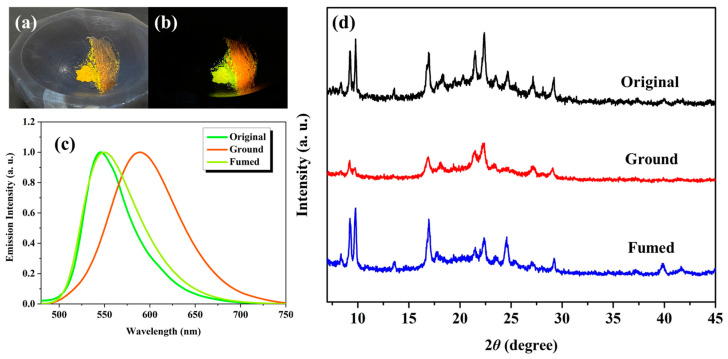
Photographic images of the as-prepared NBDNI powder and the ground NBDNI powder under room light (**a**) and 365 nm UV light (**b**). The emission spectra of as-prepared, ground, and fumed powders of NBDNI (**c**). XRD patterns of as-prepared, ground, and fumed powder samples of NBDNI (**d**).

As mentioned, when preparing the acid-responsive fluorescent test strip of NBDNI, we found that the fluorescence colors of crystalline NBDNI and amorphous NBDNI solids showed green and yellow, respectively. This phenomenon means the mechanochromism of NBDNI should be possible. As shown in Figure 9a,b, the as-prepared NBDNI powder was a yellow solid under sunshine and emitted bright green fluorescence under UV light. After simply grinding with an agate mortar, the solid NBDNI became an orange powder with weak orange emission, whereas, after fuming with the dichloromethane vapor, the bright yellow fluorescence at 550 nm appeared, which was close to the as-prepared solid. The corresponding emission spectra were depicted in Figure 9c, and it was observed that the fluorescence peak of the NBDNI solid moved from 546 nm to 589 nm after the grinding process, whereas after fuming with the dichloromethane vapor, its emission exhibited at 550 nm. It was confirmed that this was a typical reversible mechanochromism phenomenon, and the contrast was enough to be distinguished easily by the naked eye. Furthermore, X-ray diffraction (XRD) analysis provides additional insights into the reversible mechanochromic fluorescent behavior of NBDNI. A comparison of diffraction patterns between as-prepared, ground, and fumed powders is depicted in Figure 9d. The as-prepared solid showed intense and sharp diffraction peaks corresponding to regular molecular stacking in the crystalline phase. After grinding of the as-prepared solid, these sharp and intense reflection peaks weakened due to force-induced destruction of intramolecular interactions and molecular packing mode. Additionally, the sharp and intense reflection peaks reappeared after fuming by dichloromethane vapor, implying the recovery of crystalline structure. Therefore, according to these results, we believe the mutual transformation between the planar state and the twisted state of NBDNI molecules in the crystalline and amorphous phases was responsible for this reversible mechanochromic behavior of NBDNI. 

## 3. Conclusions

In summary, a stimuli-responsive fluorophore NBDNI was developed based on 1,8-naphthalimide, which responded to multiple external stimuli, including polarity, acid, temperature, and mechanical force, with remarkable fluorescence changes. Relevant measurements revealed the multi-stimuli fluorescent response thanks to its AIE and TICT properties endowed with the unique molecular structure of NBDNI. Meanwhile, preliminary applications such as fluorescence thermometry and acid/base test paper have been demonstrated. We anticipate that this study will serve as inspiration for the advancement of novel stimuli-responsive luminescent materials while providing valuable insights into the relationship between fluorescence and molecular structure.

## 4. Materials and Methods

### 4.1. Materials 

Tetrakis(triphenylphosphine)palladium (95%), 4-bromine-1,8-naphthal anhydride (98%), 4-(dimethylamino) phenylboronicacid-pinacolester (96%), and N-butylamine (95%) were purchased from Energy Chemical Co., Ltd. (Jiahua, China) and used directly. Polystyrene, polymethyl methacrylate, and polyvinyl chloride were used as commercialized reagents directly. Tetrahydrofuran (AR, Kermel Company (Colmar, France), 99%) was refluxed with metal sodium and then distilled out before use. Triethylamine (AR, 99%), hydrochloric acid (35%), N,N′-dimethylformamide (AR, 99%), potassium carbonate (97%), ethyl acetate (AR, 99%), petroleum ether (AR, 99%), dioxane (AR, 99%), hexane (AR, 99%), tetrachloromethane (AR, 99%), toluene (AR, 99%), chlorobenzene (AR, 99%), and chloroform (AR, 99%) were purchased from Tianjin Fuyu Fine Chemical Company (Tianjin, China).

### 4.2. Instrumentations and Methods

The ^1^H and ^13^C nuclear magnetic resonance (NMR) spectra were measured with a Bruker ARX 400 MHz spectrometer, and CDCl_3_ or CF_3_COOD was used as a solvent. Proton chemical shifts are reported in ppm downfield from tetramethylsilane (TMS). The absorption spectra were taken on an Agilent Spectrum Carry 100 spectrophotometer, and the background base lines were measured in pure solvents, respectively. The fluorescence spectra were taken on a PTI Qm 40 luminescence spectrometer, and the slit width was set as 5 nm for excitation and emission, respectively. The fluorescence quantum yield absolute values and lifetimes were obtained using an Edinburgh FLS1000 fluorescence spectrometer with an integrating sphere, and the slit width was set as 2.2 nm and 0.22 nm for excitation and emission, respectively. The powder X-ray diffraction was measured with Bruker D8 Discover, the scanning scope was set as 5° to 50°, and the scanning rate was set as 5°/min.

## Figures and Tables

**Table 1:** Photophysics properties of NBDNI in different solvents.

Solvents	λ_ab_ (nm)	ε $\left(10^{3}\;M^{-4}\cdot {cm}^{-1}\right)$	λ_em_ (nm)	τ (ns)	Stokes Shift (nm)	Ø (%)
Hexane	403	18.9	476	4.03	73	61.7
CCl_4_	411	13.4	499	4.05	88	58.3
Tol	419	16.6	542	5.38	123	32.6
DOX	417	15.9	567	4.85	150	25.6
PhCl	430	15.6	579	6.67	149	24.2
CF	431	15.9	585	7.06	154	19.3
THF	424	16.0	619	-	195	16.4
EtOH	432	15.4	673	-	241	-

λ_ab_: maximum absorption wavelength; ε: molar absorption coefficient; λ_em_: maximum emission wavelength; τ: fluorescence lifetime; Stokes shift: the difference of λ_ab_ and λ_em_; Ø: fluorescence quantum yield absolute values. “-” means the sign was too weak to determine.

## Data Availability

Data are contained within the article and Supplementary Materials.

## Supplementary Materials

The following supporting information can be downloaded at: https://www.mdpi.com/article/10.3390/nano14151255/s1, Figure S1: The ^1^H NMR (a) and ^13^C NMR (b) spectra of NBDNI; Figure S2: The normalized UV–Vis absorption spectra of 1 × 10^−5^ M NBDNI in toluene (15~80 °C). Table S1. Photophysics properties of NBDNI solid.
