# Supplementary material for: Multi-Stimuli-Responsive Fluorescent Molecule with AIE and TICT Properties Based on 1,8-Naphthalimide
Source: Nanomaterials (Basel). 2024 Jul 27;14(15):1255. doi: 10.3390/nano14151255 (PMC11314401; doi:10.3390/nano14151255)
Supplement: Supplementary file 1 [file nanomaterials-14-01255-s001.zip › nanomaterials-3102895-supplementary.pdf]

Supporting Information

Multi-stimuli responsive fluorescent molecule with AIE and TICT properties based on 1,8-naphthalimide

Yan Yu, Na Qiang, Zhu Liu, Ming Lu, Yuqiu Shen, Jiao Zou, Jinyu Yang * and Guocong Liu *

School of Chemistry and Materials Engineering, Huizhou University, Huizhou 516007, China; qiangna93@hzu.edu.cn (N.Q.); lz0927@hzu.edu.cn (Z.L.); reggi_lu@hzu.edu.cn (M.L.);
shenyq666@hzu.edu.cn (Y.S.); zoujiao@hzu.edu.cn (J.Z.)
* Correspondence: yangjinyu@hzu.edu.cn (J.Y.); gcl_109@hzu.edu.cn (G.L.)

Synthesis of N-butyl-4-bromine-1, 8-naphthalimide

N-butylamine (3.60 g, 49.3 mmol) was dissolved in 50 mL toluene in a dry single necked round bottom flask. The above solution was dropped into 100 mL toluene solution containing 4-bromo-1,8-naphthalene dianhydride (15.0 g, 54.2 mmol) and then the mixed solution was refluxed for 24 hours. After completing the reaction, the toluene was removed by rotary evaporation, and the residue was purified using silica gel column chromatography (SiO_2; dichloromethane/ethyl acetate 5:1) to give 9.69g of white solid obtained with a yield of 59.3%. ^{1}H NMR (400 MHz, $CDCl_3$, ppm): δ = 8.63 (dd, J = 7.3, 1.2 Hz, 1H), 8.53 (dd, J = 8.5, 1.2 Hz, 1H), 8.38 (d, J = 7.8 Hz, 1H), 8.01 (d, J = 7.8 Hz, 1H), 7.83 (dd, J = 8.5, 7.3 Hz, 1H), 4.21 – 4.13 (m, 2H), 1.77 – 1.65 (m, 2H), 1.45 (h, J = 7.4 Hz, 2H), 0.98 (t, J = 7.4 Hz, 3H).

Synthesis of N-butyl-4-(N, N′-dimethylaminobenzo)-1, 8-naphthalimide (NBDNI)

Under nitrogen atmosphere, N-butyl-4-bromine-1,8-naphthalimide (2.0 g, 6.02 mmol), 4-(dimethylamino) phenylboronicacid-pinacolester (1.35 g, 5.47 mmol), K_2CO_3 (4.9g, 35.7 mmol), and Pd $(PPh_3)_4$ (0.316g, 0.274 mmol) were dissolved in 40 mL dioxane and 10 mL deionized water, and then the mixture was refluxed for 6 hours. After cooling to room temperature, the solvent was removed by rotary evaporation. The residue was dissolved in 50 mL of dichloromethane, and washed 4 times with 20 mL deionized water. The organic layer was dried with anhydrous $MgSO_4$ powder for 8 hours. Then the filtered filtrate was concentrated using a rotary evaporator, and purified by silica gel column chromatography (SiO_2; dichloromethane/ethyl acetate=8:1). The collecting product was recrystallized in a mixed solvent of ethyl acetate and petroleum ether to obtain a yellow solid of 1.50g with a yield of 73.6%. ^{1}H NMR (400 MHz, $CDCl_3$, ppm): δ = 8.61 (d, J = 7.6 Hz, 1H), 8.41 (dd, J = 8.5, 1.2 Hz, 1H), 7.67 (dt, J = 7.3, 4.1 Hz, 2H), 7.42 (d, J = 8.7 Hz, 2H), 6.87 (d, J = 8.3 Hz, 2H), 4.25 – 4.17 (m, 2H), 3.07 (s, 6H), 1.80 – 1.68 (m, 2H), 1.46 (h, J = 7.4 Hz, 2H), 0.98 (t, J = 7.4 Hz, 3H). ^{13}C NMR (100 MHz, $CDCl_3$, ppm): δ = 164.52, 164.31,150.52, 147.48, 133.10, 130.99, 130.11, 128.99, 127.44, 126.46, 126.38, 122.92, 120.72, 112.25, 40.45, 40.24, 30.29, 20.45, 13.90.

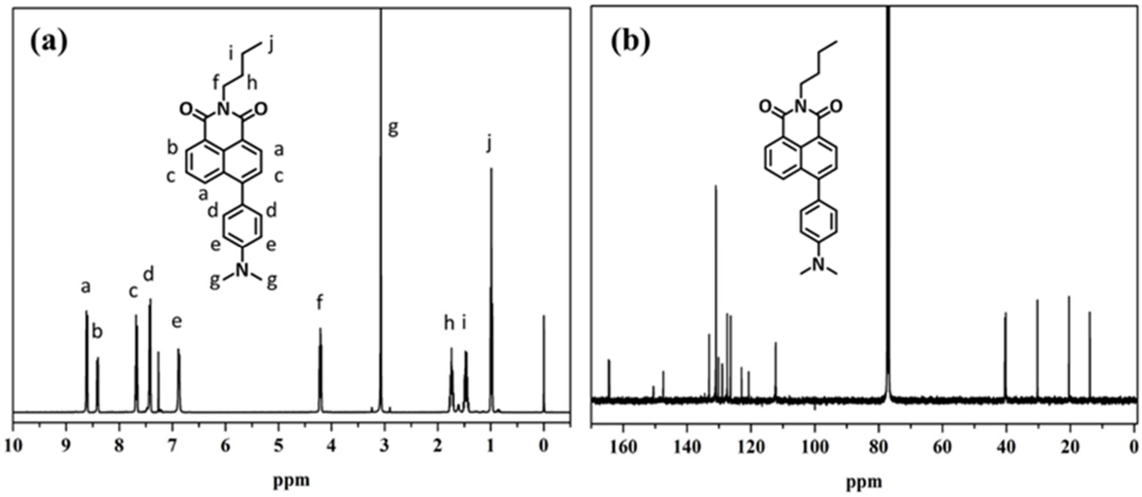

Figure S1. The ^{1}H NMR (a) and ^{13}C NMR (b) spectra of NBDNI.

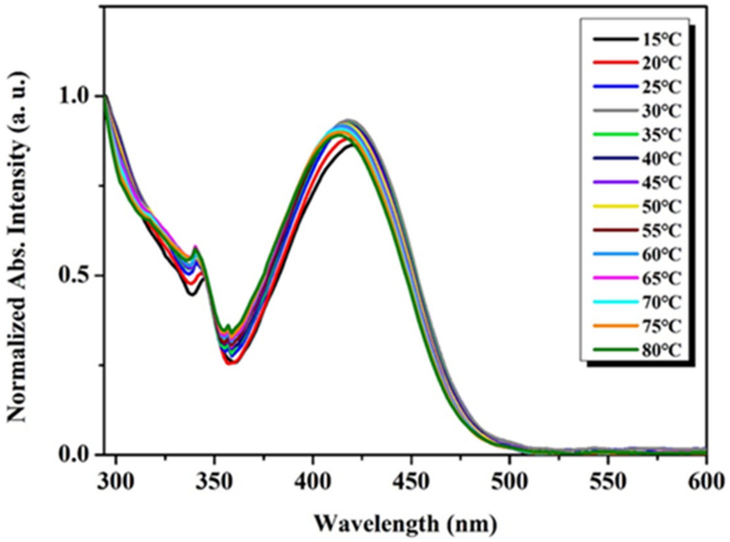

Figure S2. The normalized UV-vis absorption spectra of 1×10^{-5} M NBDNI in toluene (15 ~ 80℃).

Table S1. Photophysics properties of NBDNI solid.

	λ_{ab} (nm)	λ_{em} (nm)	τ (ns)	Stokes shifts (nm)	Ø (%)
NBDNI solid	420	546	3.97	241	15.28

λ_{ab}: maximum absorption wavelength; ε: molar absorption coefficient; λ_{em}: maximum emission wavelength; τ: fluorescence lifetime; Stokes shifts: the difference of λ_{ab} and λ_{em}; Ø: fluorescence quantum yield absolute values.
